# Equilibrium constants and protonation site for *N*-methylbenzenesulfonamides

**DOI:** 10.3762/bjoc.7.203

**Published:** 2011-12-27

**Authors:** José A Moreira, Ana M Rosa da Costa, Luis García-Río, Márcia Pessêgo

**Affiliations:** 1 CIQA Centro de Investigação em Química do Algarve, Departamento de Química e Farmácia, Faculdade de Ciências e Tecnologia, Universidade do Algarve, Campus de Gambelas, 8005-139 Faro, Portugal; 2 CIQUS Centro de Investigación Química Biológica y Materiales Moleculares, Departamento de Química Física, Facultad de Química, Universidad de Santiago, 15782, Santiago de Compostela, Spain

**Keywords:** linear free-energy relationships, *N*-methylbenzenesulfonamides, protonation equilibrium

## Abstract

The protonation equilibria of four substituted *N*-methylbenzenesulfonamides, X-MBS: X = 4-MeO (**3a**), 4-Me (**3b**), 4-Cl (**3c**) and 4-NO_2_ (**3d**), in aqueous sulfuric acid were studied at 25 °C by UV–vis spectroscopy. As expected, the values for the acidity constants are highly dependent on the electron-donor character of the substituent (the p*K*_BH+_ values are −3.5 ± 0.2, −4.2 ± 0.2, −5.2 ± 0.3 and −6.0 ± 0.3 for **3a**, **3b**, **3c** and **3d**, respectively). The solvation parameter m* is always higher than 0.5 and points to a decrease in the importance of solvation on the cation stabilization as the electron-donor character of the substituent increases. Hammett plots of the equilibrium constants showed a better correlation with the σ^+^ substituent parameter than with σ, which indicates that the initial protonation site is the oxygen atom of the sulfonyl group.

## Introduction

**Scheme 1:**
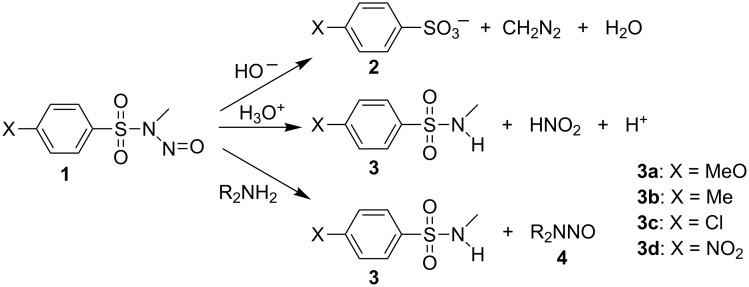
Reaction of *N*-methyl-*N*-nitrosobenzenesulfonamides **1** with nucleophiles.

Having a knowledge of the protonation equilibrium constants for *N*-methylbenzenesulfonamides **3** is fundamental to achieve a correct understanding of their reactivity, that is to say that the referred constants can be used to estimate the values of the protonation constants for *N*-methyl-*N*-nitrosobenzenesulfonamides **1**. This information, not yet experimentally available, is of crucial importance in the studies of the nitroso-group transfer mechanism from **1**. Such compounds react with a variety of nucleophiles: In the presence of HO^−^ or EtO^−^, which attack their SO_2_ group, decomposition to afford diazomethane occurs [1–2]. In acidic medium, they undergo denitrosation to the corresponding *N*-methylbenzenesulfonamides **3** [3–4], as is common with other *N*-nitrosamines. However, unlike with nitrosamines and nitrosoureas, nucleophilic attack by amines at the N=O group affords nitrosamines **4** [5] (Scheme 1). They are also known to be capable of nitroso-group transfer to form nitrosyl complexes [6–7]. Increasing attention is being paid to the chemistry of nitrosamines owing to the toxicity [8–9] and carcinogenic [10–11], mutagenic [12–14], and teratogenic [15–16] properties of these compounds.

The acidity of organic molecules is one of the most relevant factors determining their reactivity. Nevertheless, the values of the protonation and deprotonation equilibrium constants are generally difficult to obtain. This is due to the difficulties in the definition of the acidity scales and in the interpretation of the experimental data.

In diluted acid, p*K*_BH+_ can be easily evaluated by measuring the ionization ratio *I* = [BH^+^]/[B] and the proton concentration in the medium. However, in strongly acidic solutions, the ability of the medium to protonate a weak base largely exceeds the formal concentration of hydronium ions, due to the medium-induced effects in the activity coefficients of the different species involved in the equilibrium. Historically, there were two approaches to the analysis of such effects in strongly acidic media.

The first approach emphasizes the acidity of the medium and is derived from Hammett’s approach, proposed in 1932 [17] in order to achieve an acidity measure contiguous to the pH scale, defined for dilute aqueous solutions. With this purpose, Hammett defined the so-called “Hammett acidity function”, *H*_0_, which is no more than a measure of the deviation, relative to ideality, provoqued by the changes in the medium as the acid concentration increases.

Time has proved that Hammett’s methodology is only applicable to similar classes of compounds [18–19]. In reality, during the 1950’s, a variety of acidity constants for different kinds of bases, such as tertiary amines (*H*_0_’’’) [20], amides (*H*_a_) [21], carbinoles (*H*_R+_) [22], and indoles (*H*_I_) [23], among others [19,24], were developed.

The second approach to the problem considers that variations in the equilibrium or rate constants in aqueous acidic mixtures may be described by a free-energy linear correlation. This approach was developed by Bunnett and Olsen [25–26] according to the suggestion of Grunwald [27] and Kresge [28], has been broadly used [29–37], and was reviewed by Bagno, Scorrano and More O’Ferrall in 1987 [38].

In order to use Grunwald’s formalism [27] to account for the effects of the medium on acid–base equilibria, a reference equilibrium must be chosen, to which the dependence on the acidity of any other equilibrium is compared. In Equation 1, *K* and K* are, respectively, the equilibrium constants of the reaction under study and of the reference reaction, and δ_M_ accounts for the effects of changes in the medium (i.e., in the concentration of the strong acid).

[1]
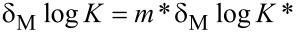


Equation 1 may be rewritten in the more familiar form of Equation 2, where *K*_0_ and *K*_0_* are the equilibrium concentration ratios in a reference solvent, which in the case of reactions in aqueous acidic media is normally water.

[2]
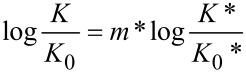


If we denote log K*/*K*_0_* as –*X*, this equation becomes

[3]
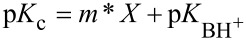


where *K*_c_ is the experimental classical ionization constant and *K*_BH+_ the thermodynamic ionization constant in water. According to the interpretation of Bagno and Scorrano [38], m* is a measure of the cation (the protonated base, BH^+^) solvation, that is, a solvation coefficient. So, the strength of a weak base is determined by its p*K*_BH+_ in the reference solvent, usually water, and by its solvation coefficient in acidic medium. These parameters are the intercept and the slope of Equation 3. The choice of water as the reference solvent and of 4-nitroaniline as reference base, renders m* = 0 for the pair H_3_O^+^/H_2_O and m* = 1 for pairs formed by anilinium ions and the respective aniline.

## Results and Discussion

The determination of the classical equilibrium constant, *K*_c_, requires knowledge of the ionization ratio *I* = [BH^+^]/[B]. Usually this is obtained by UV–vis spectroscopic measurements, as *I* relates to the absorbance according to Equation 4

[4]
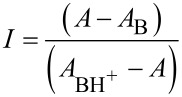


where *A*, *A*_B_ and *A*_BH+_ are the absorbances of the solution, of the free base and of its conjugated acid, respectively.

**Figure 1:**
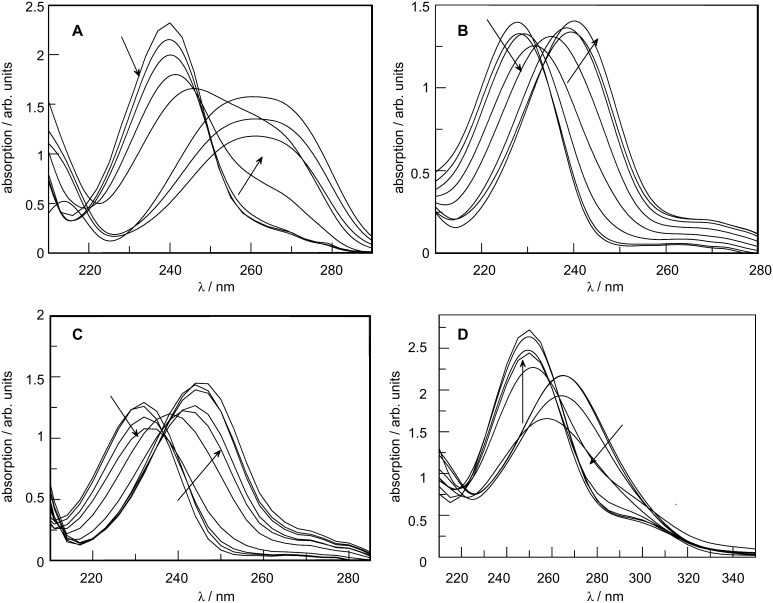
Absorption spectra at 25 °C of 5 × 10^−5^ M aqueous sulfuric acid solutions of: (A) **3a**, (B) **3b**, (C) **3c**, and (D) **3d**. Acid concentration varies between 0 and 97% (w/w).

Figure 1 presents the spectra of the four benzenesulfonamides (**3a**–**d**) under study, in which a visible change occurs as the substrates protonate.

The most striking observation related to the above spectra is the absolute lack of isosbestic points, which arises from the shift in the *n* → π* absorption bands of the sulfonamides as the acid concentration increases. In order to eliminate this effect, the spectra must be treated by the characteristic vectors analysis (CVA) method [39].

This analysis requires the construction of a matrix of absorbances at different wavelengths and different acid concentrations, from which an average absorbance matrix and a number of characteristic vectors that allegedly contain all the information of the original data are obtained (Equation 5).

[5]
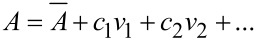


In most cases, the original data are reproduced with 99% accuracy from two vectors only, in which the first accounts for 94–96% of the variation and the second for the remaining 3–6%. Based on our chemical intuition, we associate the first to the protonation process and the second to the medium effect [40].

**Figure 2:**
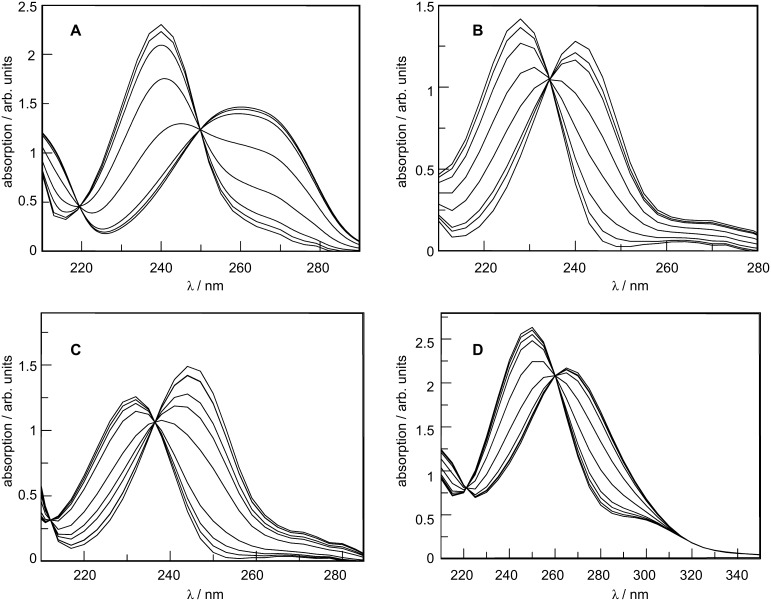
post-CVA absorption spectra at 25 °C of 5 × 10^−5^ M aqueous sulfuric acid solutions of: (A) **3a**, (B) **3b**, (C) **3c**, and (D) **3d**. Acid concentration varies between 0 and 97% (w/w).

Figure 2 shows the spectra obtained after application of the CVA method (considering that the protonation effect is given by the ν_1_ vectors). The data was treated according to Simonds original algorithm [39] implemented on Mathcad [41].

**Figure 3:**
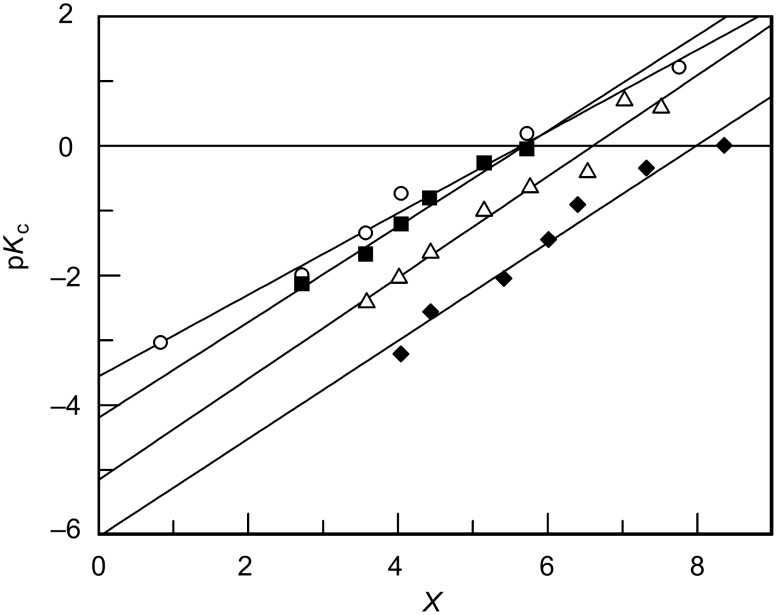
Plot of p*K*_c_ against *X* for the protonation equilibrium of (circles) **3a**, (squares) **3b**, (triangles) **3c** and (diamonds) **3d** in aqueous sulfuric acid solutions. [X-MBS] = 5 × 10^−5^ M, 25 °C.

The values for the ionization ratio are determined from Equation 4. The composition of the sulfuric acid solution when *I* = 1 that corresponds to a degree of protonation of 50% can be easily calculated and is namely 65.2, 68.2, 74.0 and 80.6% sulfuric acid (w/w) for compounds **3a**, **3b**, **3c** and **3d**, respectively. [H^+^] and *X* values for each sulfuric acid concentration were calculated by interpolation of values from reference [38]. Since p*K*_c_ = −log [H^+^] + log *I*, data may now be fitted to Equation 3 in order to obtain m* and p*K*_BH+_ values (Figure 3).

From the results presented in Table 1 it is evident, as expected, that there is an increase in the acidity constant with the electron-withdrawing character of the substituents. The solvation parameter m* is higher than 0.5 in all cases and also increases with the electron-withdrawing character of the substituents in the ring, which indicates a decrease in the solvation degree [42].

**Table 1:** Obtained p*K*_BH+_ and m* values for different *para*-substituted *N*-methylbenzenesulfonamides.

X-MBS	p*K*_BH+_	m*

**3a**	−3.5 ± 0.2	0.63 ± 0.03
**3b**	−4.2 ± 0.2	0.74 ± 0.05
**3c**	−5.2 ± 0.3	0.78 ± 0.06
**3d**	−6.0 ± 0.3	0.76 ± 0.06

**Scheme 2:**
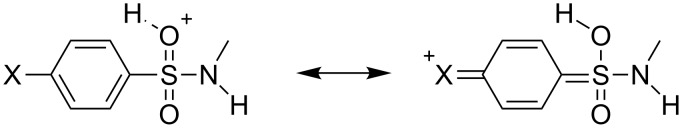
Resonance stabilization of O-protonated *N*-methylbenzenesulfonamides, **3**.

These results allow us to make some conjectures about the protonation site. Considering that the SO_2_ group prevents resonance between the nitrogen atom and the ring, the dependence of the acidity constant on the electronic character of the substituents seems too overwhelming to support protonation on the nitrogen atom. Being so, it is more likely that the protonation occurs on the sulfonyl oxygen atom, as such a structure may present resonance with the electron-donor substituents (Scheme 2).

**Figure 4:**
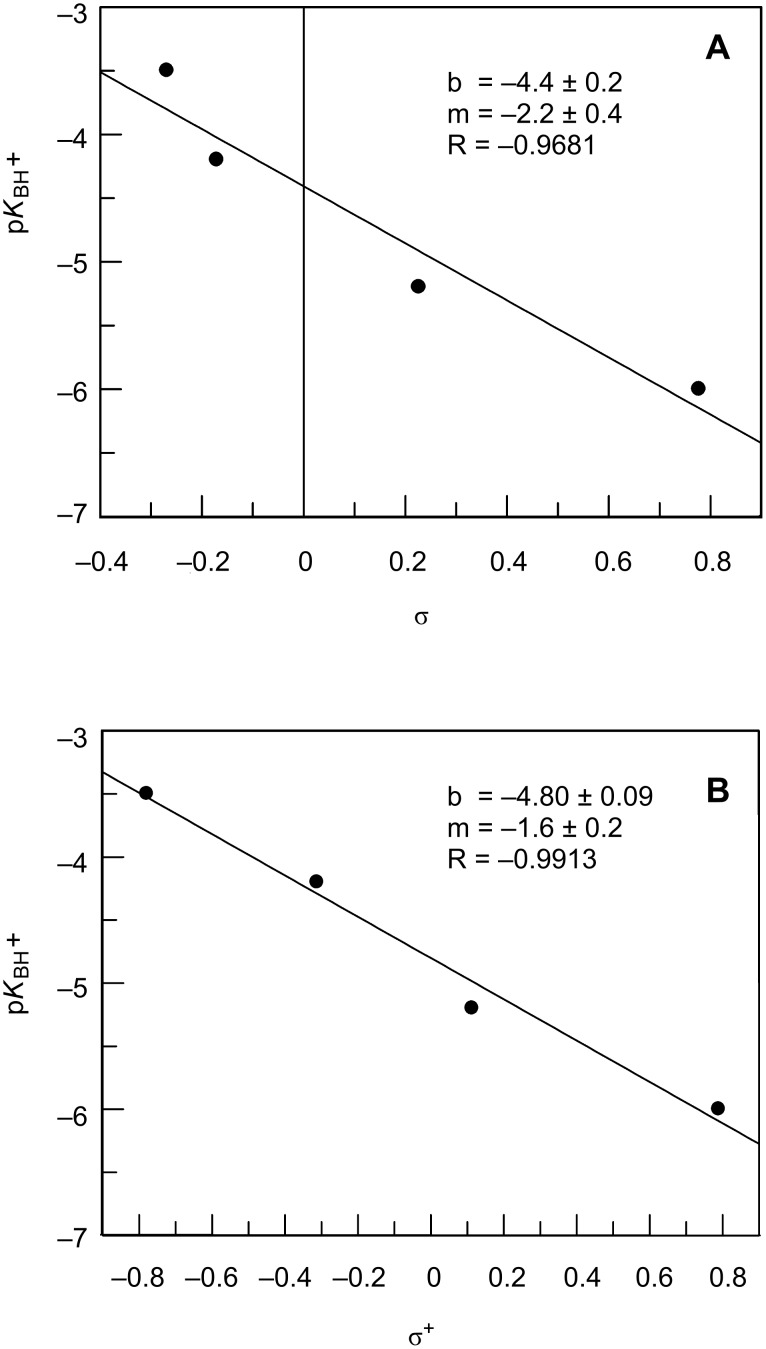
Correlation between p*K*_BH+_ and (A) σ or (B) σ^+^ for compounds **3a**–**d**.

The fact that p*K*_BH+_ correlates better with σ^+^ (R = 0.9913) than with σ (R = 0.9681) also indicates protonation on the oxygen atom (Figure 4). Nevertheless, the curvature of the σ Hammet plot could be ascribed to a change in the protonation site from oxygen, on the compounds carrying the more electron-donating substituents, to nitrogen, for those with the more electron-withdrawing substituents. However, if this were the case, the curvature in the correlation with σ^+^ would be more pronounced. Moreover, the solvation parameter m* values found also seem to be compatible with oxygen protonation, since for oxygen bases these values range from 0 to 0.7 but for nitrogen bases lie around unity [38]. In fact, although Menger and Mandell [43] concluded that *N*-methyl-5-chloro-1,2-benzisothiazoline 1,1-dioxide in fluorosulfonic acid protonated on the nitrogen atom, Chardin and co-workers [44] showed that protonation of sulfonamides occurred on the oxygen atom.

**Scheme 3:**
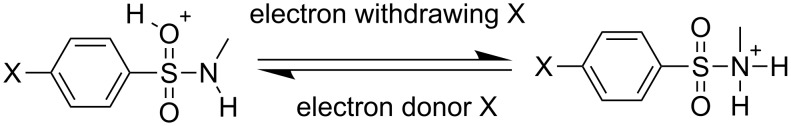
Tautomeric equilibrium between N- and O-protonated forms of *N*-methylbenzenesulfonamides, **3**.

Still, a possibility that should not be discarded is the existence of a tautomeric equilibrium between the N*-* and O*-*protonated structures, the latter having a greater relevance for the sulfonamides with electron-donor groups (Scheme 3).

## Conclusion

The protonation equilibrium constants (p*K*_BH+_) for the *para*-substituted *N*-methylbenzenesulfonamides **3a–d** in aqueous sulfuric acid were obtained from spectrophotometric measurements. Treatment of the spectra by the characteristic vectors analysis (CVA) method, in order to compensate for the shift in the *n* → π* absorption bands of the sulfonamides as the acid concentration increases, was necessary. The values obtained were seen to increase with the electron-withdrawing character of the substituents.

The solvation parameter (m*) values point to a decrease in the degree of solvation as the electron-withdrawing character of the substituents increases and to protonation on the oxygen atom.

The correlation between p*K*_BH+_ and σ^+^ also indicates oxygen protonation, although the existence of a tautomeric equilibrium between the N- and O-protonated forms cannot be ruled out.

## Experimental

### Synthesis of *N*-methylbenzenesulfonamides

The *N*-methylbenzenesulfonamides **3a–d** were prepared from the reaction of the parent benzenesulfonyl chlorides with methylamine [3,45].

### Preparation of acid solutions

Acid solutions were always prepared by weighing the appropriated amount of commercial H_2_SO_4_ (98%, Aldrich), which was then carefully diluted in water, and small aliquots of the mixture were then titrated with NaOH solution. The resulting molarities were converted to weight percents by using the conversion table published in the CRC Handbook of Chemistry and Physics [46]. The concentrations of the acid solutions were double checked by measuring the densities of the solutions.

All dilutions were made in an ice bath, with careful mixing to prevent the risk of a sudden temperature rise. The solution was then allowed to stand in a water bath at 20 °C and the final volume in the volumetric flask was adjusted.

### Spectroscopic measurements

Solutions of **3a–d** (5.0 × 10^−5^ M) were prepared by adding a small amount, typically 30 µL, of a stock solution to 10 mL of the sulfuric acid solution. UV spectra were recorded in a Varian Cary 100 equipped with a thermostated cell holder. All measurements were made in quartz cells with a 1 cm light path, at 25 °C, and the spectra were run against a solution with the same concentration of sulfuric acid as that of the *N*-methylbenzenesulfonamide solution.
